# Sleep Disorders in Leucine-Rich Glioma-Inactivated Protein 1 and Contactin Protein-Like 2 Antibody-Associated Diseases

**DOI:** 10.3389/fneur.2020.00696

**Published:** 2020-07-30

**Authors:** Nan Lin, Honglin Hao, Hongzhi Guan, Heyang Sun, Qing Liu, Qiang Lu, Liri Jin, Haitao Ren, Yan Huang

**Affiliations:** Department of Neurology, Peking Union Medical College Hospital, Beijing, China

**Keywords:** sleep disorders, polysomnography, states dissociate, LGI1 antibody encephalitis, Caspr2 antibody-associated diseases

## Abstract

**Objective:** Sleep disorders are common in voltage-gated potassium channel complex antibody (VGKC-Ab) diseases. The aim was to investigate the sleep disturbances and polysomnography (PSG) characteristics in patients with VGKC-Ab-associated diseases.

**Methods:** Twenty-seven patients with leucine-rich glioma-inactivated protein 1 antibody (LGI1-Ab) encephalitis, seven patients with contactin protein-like 2 antibody (Caspr2-Ab)-associated diseases, and 14 healthy controls with at least one PSG or actigraphy recording were recruited at Peking Union Medical College Hospital from January 2014 to July 2019.

**Results:** Sleep disorders including insomnia, hypersomnia, rapid eye movement (REM) sleep behavior disorder (RBD), periodic limb movements in sleep (PLMS), agrypnia excitata, and obstructive sleep apnea syndrome were observed. Twenty-one PSG recordings from patients with LGI1-Ab encephalitis demonstrated a decrease in total sleep time (TST) (median 365.5, range 184.5–495.5 min), sleep efficiency (70.0%, 47–92%), N3 sleep (9.7%, 0–32.9%), and REM sleep (9.9%, 0.4–27.9%). Of five patients with Caspr2-Ab-associated diseases, TST was found to be 329.5 (167.0–377.5 min), and sleep efficiency was found to be 61.7% (34.6–71.7%). The percentage for N3 and REM sleep was found to be 15.0% (0–34.6%) and 12.7% (0–22.2%), respectively. Both RBD and PLMS were observed more frequently in patients with LGI1-Ab encephalitis. We identified status dissociatus (SD) in five (23.8%) patients with LGI1-Ab encephalitis and two (40%) patients with Caspr2-Ab diseases. The former is more likely to have simple limb movements rather than complex movements, which mimic the contents of their dreams. Continuous insomnia was more common in patients with Caspr2-Ab diseases than patients with LGI1-Ab encephalitis. Patients reported clinical and PSG improvements following immunotherapy.

**Conclusion:** Sleep disorders in patients with VGKC-Ab-associated diseases include decreased TST and poor sleep efficiency. Our studies provide evidence of SD in patients with LGI1-Ab encephalitis.

## Introduction

Voltage-gated potassium channels (VGKCs) are present on the membrane of neurons. VGKC complex antibodies (VGKC-Abs) were initially identified in patients with neuromyokymia (1–4) and limbic encephalitis (5, 6). The main target antigens of VGKC-complex proteins are (i) leucine-rich glioma-inactivated protein 1 (LGI1), a secreted protein that expresses abundantly in the hippocampus and the temporal cortex and binds to proteins of the ADAM (a disintegrin and metalloproteinase) family (7, 8); (ii) contactin protein-like 2 (Caspr2) that expresses in the juxtaparanodal region of myelinated axons in both brain and peripheral nerve (8, 9).

LGI1 antibody (LGI1-Ab) is mainly associated with limbic encephalitis and faciobrachial dystonic seizures (FBDSs). Caspr2 antibody (Caspr2-Ab) is associated with more diverse symptoms including neuromyokymia, Morvan's syndrome, and limbic encephalitis (10). Sleep disorders are prominent manifestations of Morvan's syndrome and also commonly seen in LGI1-Ab encephalitis. These clinical features include insomnia, rapid eye movement (REM) sleep behavior disorder (RBD), periodic limb movements in sleep (PLMS), hypersomnia, agrypnia excitata, and obstructive sleep apnea syndrome (OSAS) (11–13). However, the spectrum of sleep manifestations in LGI1-Ab and Caspr2-Ab diseases has not been systematically studied. We conducted a retrospective study to investigate sleep disturbance and polysomnography (PSG) characteristics in patients with LGI1-Ab encephalitis and Caspr2-Ab-associated diseases.

## Methods

### Patients

The study enrolled 48 participants with at least one PSG or actigraphy recording at the Peking Union Medical College Hospital (PUMCH) from January 2014 to July 2019. Twenty-seven subjects were diagnosed with LGI1-Ab encephalitis and seven with Caspr2-Ab diseases. We included 14 age-matched healthy controls who had no known history of sleep disorders or neurological illness and no known factors that may affect sleep such as shift work. All had a Pittsburgh sleep quality index ≤ 5. The demographic information, clinical features, and laboratory test data were reviewed. The diagnosis of autoimmune limbic encephalitis was based on the criteria established by Graus et al. (14): (i) subacute onset of memory deficits, seizures, or psychiatric symptoms suggesting involvement of the limbic system; (ii) T2-weighted hyperintensities of medial temporal lobes; (iii) at least one of the following: cerebrospinal fluid (CSF) pleocytosis, electroencephalogram (EEG) epileptic, or slow-wave activity; (iv) exclusion of other causes. Morvan's syndrome was defined as a combination of cognitive symptoms or seizures, peripheral nerve hyperexcitability, and dysautonomia or insomnia (15).

### Determination of Antibodies

We conducted a fixed cell-based indirect immunofluorescence test (IIFT) for LGI1 and Caspr2 Abs using Biochips (Euroimmun AG, Lübeck, Germany).

### Sleep Assessments

Sleep was evaluated by video PSG and actigraphies. Overnight video PSG monitoring was performed using the 76-channel EEG/PSG recording system (Compumedics Grael series; Australia), at the sleep laboratory of PUMCH. The minimal recording time was 7 h. The following signals were recorded: at least six EEG channels (two frontal, two occipital, and two central), two electro-oculography channels, two electromyography channels (mentalis/submentalis and tibialis), nasal airflow, respiratory efforts (thoracic and abdominal), pulse oximetry, body position, and a single-led electrocardiogram. The results of PSGs were evaluated by experienced sleep specialists blinded to clinical diagnosis, on the basis of standard scoring practices (16). We analyzed the following sleep parameters: total sleep time (TST), sleep onset latency, sleep efficiency, the percentages of respective sleep stages, limb movement in sleep (LMS) index, micro-arousal index, and apnea–hypopnea index (AHI).

Sleep monitor by actigraphy was recorded using the ActiGraph (Compumedics, Australia), placed on the non-dominant wrist, for seven consecutive days at PUMCH. Data were analyzed by experienced evaluators. For analysis of sleep–wake differentiation, we used the ActiLife 6 software provided by the manufacturer (Compumedics ActiGraph). The software uses the algorithm by Cole et al., which was validated in an adult population (17). The 24-h TST was measured. All sleep assessments were performed during the acute phase of the diseases. Most PSG and actigraphy recordings were performed during treatment. All assessments were performed before marked clinical improvements.

Before sleep assessments, patients and bed partners were specifically asked about sleep complaints including insomnia, excessive daytime sleepiness, limb movements, dream enactment behavior, or snoring. Sleep specialists also evaluated the symptoms by video during PSG monitoring.

Patients who had trouble staying awake while driving, eating, or engaging in social activities were considered to have excessive daytime sleepiness. Daytime sleepiness was assessed by free interview. Dream enactment behavior was defined as nocturnal vocalizations and limbs or trunk movements with dream ideation reported by a bed partner. Status dissociatus (SD) was reported as a polysomnographic trait, which was characterized by (i) lack of the conventional features of non-REM (NREM) sleep with the disappearance of spindle and delta activities; and (ii) unstable REM sleep appearing in short recurrent episodes, isolated, or mixed with NREM potentials (18). The most severe type of SD, associated with nearly continuous motor overactivation and peculiar dream-like behavior, was known as agrypnia excitata. Agrypnia excitata had been reported in Morvan's syndrome, familial insomnia, and alcohol withdrawal syndrome (18, 19). Patients who had difficulty in initiating sleep, difficulty maintaining sleep, waking up too early, or TST of <360 min resulting in daytime sleep impairment were considered to have insomnia. Continuous insomnia was defined as no sleep for more than 2 days. Clinical improvements were based on patient subjective feelings, family observations, and neurological examinations by clinicians.

### Statistical Analysis

Continuous non-normal distribution data, such as PSG data, were described as medians with range. Mann–Whitney *U* test was performed for comparisons between two groups. The frequency data between two groups were compared by Fisher's exact test. All the tests were two-tailed, and statistical significance was set at *p* = 0.05.

## Results

A total of 120 patients were diagnosed with LGI1-Ab encephalitis at PUMCH. Fifty-one (42.5%) of these patients presented with sleep disorders. Twenty-four patients were diagnosed with Caspr2-Ab-associated diseases, of whom 11 (45.8%) patients had sleep disturbances. Data from all participants with detailed sleep assessments were reviewed as below.

### LGI1 Antibody Encephalitis

Twenty-seven patients with LGI1-Ab encephalitis underwent PSG or actigraphy recordings. All patients had positive serum/CSF LGI1-Ab, but none had positive Caspr2-Ab in serum or CSF. Eighteen (66.7%) patients were male. Their median age was 63 years (range 36–77 years). All patients met the criteria of limbic encephalitis. Almost all patients developed memory deterioration (96.3%) and seizures (85.2%). Other clinical symptoms included psychiatric disorders (66.7%), FBDSs (66.7%), dysautonomia (51.9%), hyponatremia (48.1%), and consciousness disorder (7.4%) (Table 1). CSF studies showed mildly elevated protein in five patients (0.47–0.63 g/L) and elevated white cells (8 cells/μl) in one case. One patient had gastric cancer. One patient had a hyper-metabolism mass between the left subscapularis and anterior serratus muscles in PET/CT. This patient refused biopsy and was lost to follow-up.

**Table 1:** The clinical manifestations of patients with LGI1-antibody encephalitis and Caspr2-antibody-associated diseases.

	**LGI1 (*n* = 27)**	**Caspr2 (*n* = 7)**
Gender, M:F	18:9	5:2
Age	63 (36–77)	59 (24–70)
Hospital days	21 (8–47)	29 (27–45)
Seizures	23 (85.2%)	2 (28.6%)
Psychiatric disorders	18 (66.7%)	4 (57.1%)
FBDSs	18 (66.7%)	0
Consciousness disorder	2 (7.4%)	1 (14.3%)
Myokymia	0	7 (100%)
Memory deterioration	26 (96.3%)	3 (42.9%)
Dysautonomia	14 (51.9%)	7 (100%)
Sleep disorders	27	7
Insomnia	15 (55.6%)	7 (100%)
Limb movement	18 (66.7%)	5 (71.4%)
Dream enactment behavior	5 (18.5%)	4 (57.1%)
Excessive daytime sleepiness	12 (44.4%)	3 (42.9%)
Sleep talking	3 (11.1%)	0
Snoring	1 (3.7%)	0
Ataxia	1 (3.7%)	2 (28.6%)
Hyponatremia	13 (48.1%)	4 (57.1%)
Abnormal PET/CT	14/17 (82.4%)	2/4 (50%)
Abnormal MRI	19 (70.4%)	0 (0/6)
Elevated CSF white cells	1 (3.7%)	2 (28.6%)
Elevated CSF proteins	5 (18.5%)	2 (28.6%)
Tumor	2 (7.4%)	0

*LGI1, leucine-rich glioma-inactivated protein 1; Caspr2, contactin protein-like 2; FBDSs, faciobrachial dystonic seizures; CSF, cerebrospinal fluid*.

Nineteen (70.4%) participants showed abnormal brain MRI finding. Eighteen cases had mesial temporal lobe lesions, and two had basal ganglion lesions. ^18^F-FDG PET/CTs were available for 17 patients. Among these, 82.4% patients demonstrated an abnormal metabolic pattern involving medial temporal lobes (*n* = 12), basal ganglia (*n* = 11), cortex (*n* = 2), and thalamus (*n* = 1). Twenty patients completed EEG recordings. Among these, 70% had abnormal patterns including 11 patients with epileptiform discharge, seven with focal or multifocal slow waves, and three with diffused slow waves.

All 27 patients developed new-onset or worsened sleep disturbances during the acute phase of encephalitis. Abnormal LMS presented in 18 (66.7%) patients. Five of these patients had dream enactment behaviors. Other sleep disturbances include insomnia (55.6%), excessive daytime sleepiness (44.4%), sleep talking (11.1%), and snoring (3.7%) (Table 1).

Twenty-one patients underwent PSG recordings (Table 2). The medium of TST was 365.5 (184.5–495.5) min, and sleep efficiency was 70.0% (47–92%). The percentages of the respective sleep stages were 20.1% for N1 (4–86%), 51.0 % for N2 (0.7–73.2%), 9.7% for N3 (0–32.9%), and 9.9% for stage REM (0.4–27.9%). The LMS index was 62.6/h (range 0–320.8/h).

**Table 2:** PSG results of patients with LGI1-Ab encephalitis and healthy controls.

	**LGI1**	**Controls**	***p***
	**(*n* = 21)**	**(*n* = 14)**	
Age	63 (36–74)	57.5 (48–67)	0.359
Gender (M:F)	14:7	9:5	1.000
Sleep onset latency (min)	15 (0–74)	12.5 (0–38)	0.752
REM sleep latency (min)	69.5 (1.5–279)	86.5 (7.5–345)	0.164
Sleep period time (min)	522.0 (375–586.5)	534.7 (491–672.5)	0.077
Total sleep time (min)	365.5 (184.5–495.5)	430.0 (324–552.5)	0.018
Sleep efficiency (%)	70.0% (47–92%)	75.4% (61.5–93.5%)	0.040
REM (%)	9.9% (0.4–27.9%)	16.1% (5.8–23.8%)	0.044
N1 (%)	20.1% (4–86%)	12.1% (6.1–19.5%)	0.008
N2 (%)	51.0% (0.7–73.2%)	52.8% (42.8–58.5%)	0.654
N3 (%)	9.7% (0–32.9%)	19.5% (8.3–23.4%)	0.006
Total wake time (min)	118.0 (37–266)	107.7 (35–202.5)	0.210
Micro-arousal index	31.5 (6.8–59.4)	23.3 (0–52)	0.616
Limb movement in sleep index	62.6 (0–320.8)	12.7 (0–49.5)	0.001

*PSG, polysomnography; LGI1, leucine-rich glioma-inactivated protein 1; REM, rapid eye movement*.

Healthy control and patients with LGI1-Ab encephalitis showed no significant differences in age and gender (Table 2). Patients with LGI1-Ab encephalitis had markedly decreased TST (*p* = 0.018) and sleep efficiency (*p* = 0.040). We also found a decrease in the percentage of REM sleep (*p* = 0.044) and N3 sleep (*p* = 0.006) and an increase in the percentage of N1 sleep (*p* = 0.008) and LMS index (*p* = 0.001).

PSG demonstrated REM sleep without atonia (RWA) in eight patients. Five of the patients had dream enactment behaviors and were diagnosed with RBD. Other abnormal findings included PLMS in 12 (57.1%) patients and OSAS (9.5%) in two patients (AHI ≥ 5/h) according to the *International Classification of Sleep Disorders* (3rd edition). The frequencies of FBDSs, RWA, PLMS, and epileptiform discharges were not significant based on PSG parameters including sleep efficiency, TST, and the percentages of the respective sleep stages (Supplement 1).

**Figure 1:**
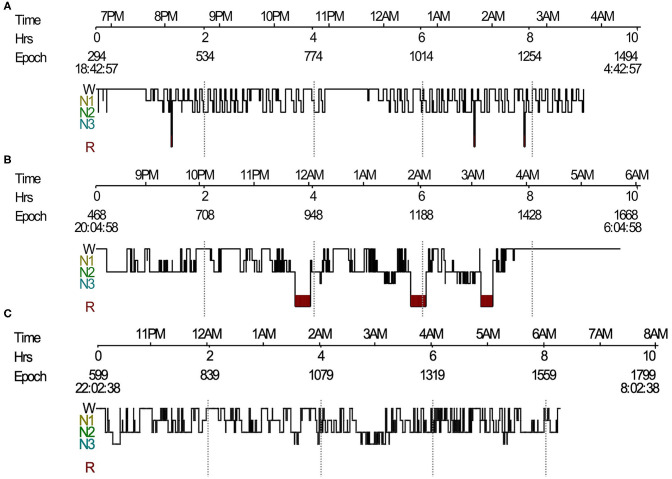
Sleep histogram. **(A)** Sleep histogram in a patient with LGI1-antibody encephalitis revealed severe impaired sleep structure, short REM sleep episodes mixed with NREM sleep, and no N3 sleep. **(B)** Follow-up PSG showed identified sleep architecture and stable REM sleep. **(C)** Sleep histogram in a Morvan's syndrome patient showed absence of REM sleep and frequent shifts between wake and NREM sleep. LGI1, leucine-rich glioma-inactivated protein 1; REM, rapid eye movement; NREM, non-rapid eye movement; PSG, polysomnography.

**Figure 2:**
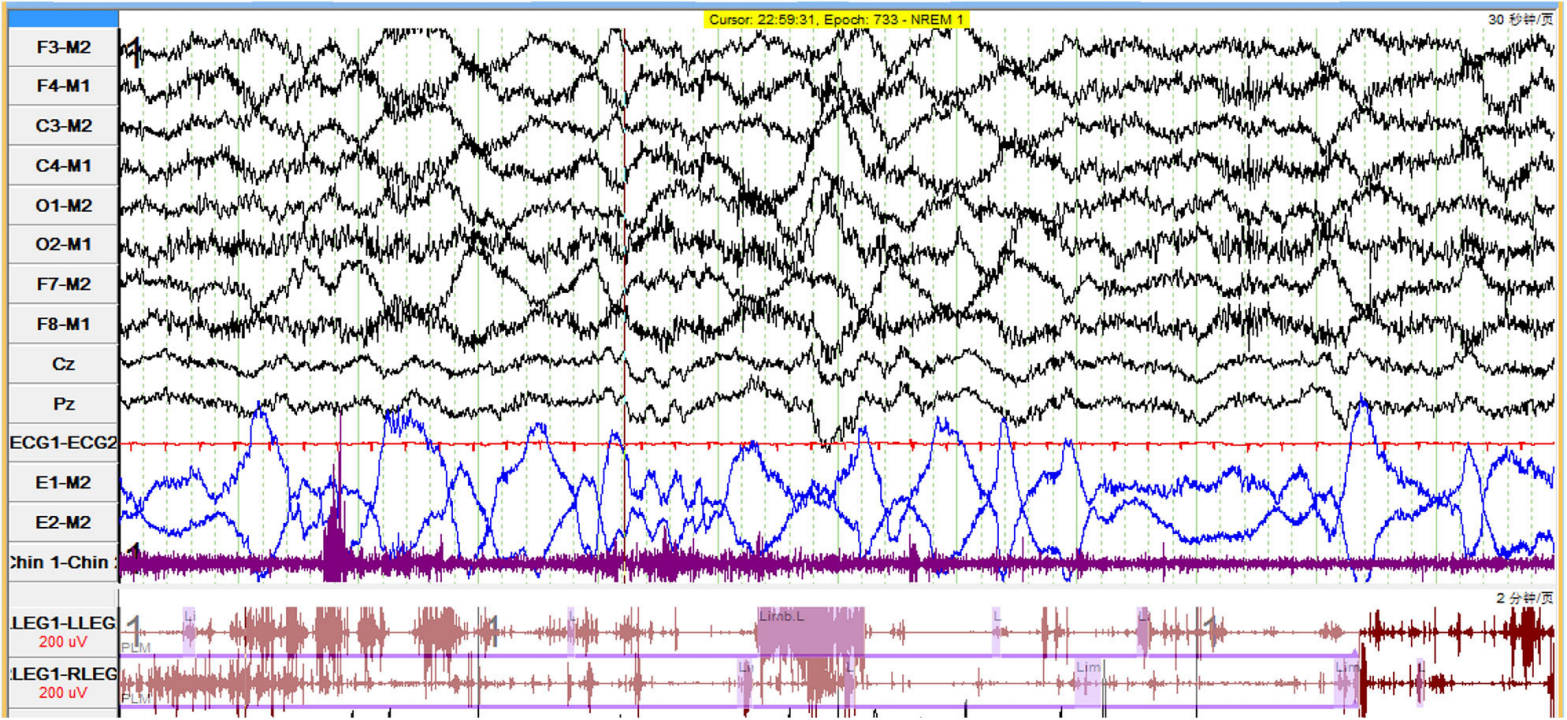
Polygraphic recording in a patient with LGI1-antibody encephalitis who had status dissociatus showed dominated slow eye movements interspersed with rapid eye movements. LGI1, leucine-rich glioma-inactivated protein 1.

We found a complete breakdown of state-determining boundaries and severe sleep fragmentation in five patients. Their PSG results showed frequent shifts between wake, N1 and N2 sleep stages, and REMs frequently intermixed with slow eye movements (Figures 1A, 2), which were consistent with the features of SD. Three (60%) patients lacked N3 sleep with scanty or absent sleep spindles. Four (80%) patients lacked stable REM sleep. Of these five patients with SD, TST was 356.0 (267–495.5) min, and sleep efficiency was 68% (50–91%). None had complex movements mimicking the contents of their dreams. Instead, we found markedly increased limb movements in three cases (LMS index 241.6–320.8/h). Three patients had PLMS, two had RWA, and four had epileptiform discharges. None had OSAS. Patients with SD had a significant decrease of REM sleep (*p* = 0.011) and an increase of N1 sleep (*p* = 0.032), than had patients without SD (Table 3). We also observed a trend of N3 sleep decline (*p* = 0.050). Other PSG parameters and frequencies of clinical symptoms showed no significant differences.

**Table 3:** The clinical and PSG characteristics in LGI1-Ab encephalitis with status dissociatus (SD) and without SD.

	**LGI1 with SD**	**LGI1 without SD**	***p***
	**(*n* = 5)**	**(*n* = 16)**	
Gender, M:F	3:2	11:5	1
Age	66 (55–68)	60.5 (36–74)	0.445
Hospital days	28 (16–36)	18 (8–47)	0.275
Seizures	5 (100%)	13 (81.3%)	0.549
Psychiatric disorders	3 (60%)	11 (68.8%)	1
FBDSs	4 (80%)	10 (62.5%)	0.624
Consciousness disorder	0	1 (6.3%)	1
Memory deterioration	4 (80%)	16 (100%)	0.238
Dysautonomia	2 (40%)	9 (56.3%)	1
Hyponatremia	3 (60%)	6 (37.5%)	0.611
Abnormal MRI	3 (60%)	11 (68.8%)	1
Sleep onset latency (min)	15.5 (3.0–74.0)	14.3 (0–29.5)	1
Total sleep time (min)	356.0 (267.0–495.5)	366.8 (184.5–467.5)	0.905
Sleep efficiency (%)	68% (50–91%)	71% (47–92%)	0.842
REM (%)	4.3% (0.4–13.8%)	12.9% (3.9–27.9%)	0.011
N1 (%)	48.9% (14–86%)	18.3% (4–42%)	0.032
N3 (%)	0 (0–21.9%)	10.9% (0.3–32.9%)	0.050
PLMS	4 (80%)	9 (56.3%)	0.606
LMS index	241.6 (0–320.8)	60.8 (0–280.9)	0.266
OSAS	0	2 (12.5%)	1
RWA	2 (40%)	7 (43.8%)	1
Epileptiform discharges	4 (80%)	6 (37.5%)	0.149
Micro-arousal index	21.3 (15.2–292)	33.6 (6.8–366)	0.866

*PSG, polysomnography; LGI1, leucine-rich glioma-inactivated protein 1; FBDSs, faciobrachial dystonic seizures; PLMS, periodic limb movements in sleep; LMS, limb movement in sleep; OSAS, obstructive sleep apnea syndrome; RWA, rapid eye movement sleep without atonia*.

**Figure 3:**
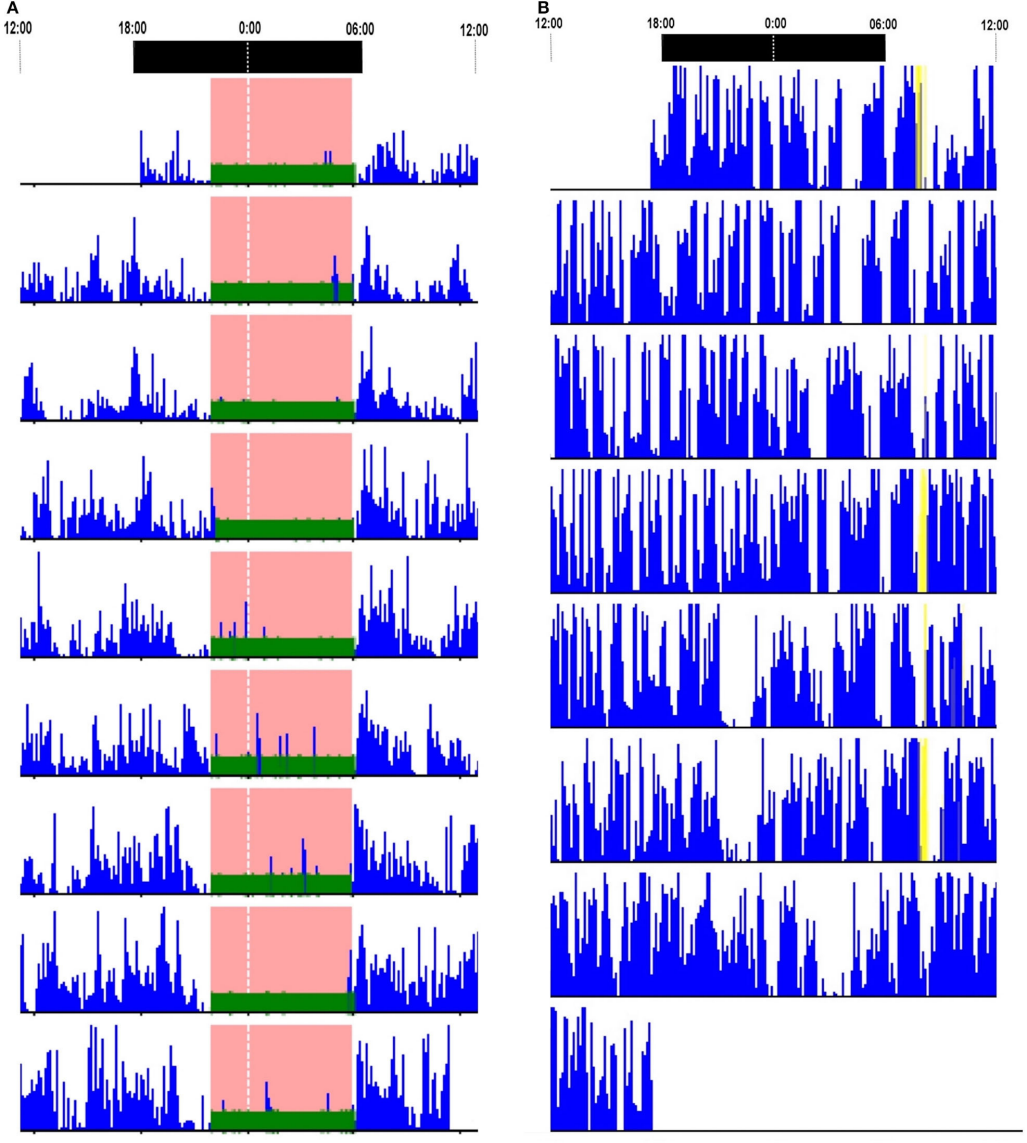
Actigraphy reports. **(A)** Actigraphy in a patient with LGI1-antibody encephalitis who had status dissociatus showed 24-h day–night motor rhythmicity. **(B)** Actigraphy in a patient with Caspr2-antibody-associated diseases showed continuous insomnia. LGI1, leucine-rich glioma-inactivated protein 1.

Ten patients underwent actigraphy studies. Continuous insomnia was observed in one patient (10%). A 24-h day–night activity rhythmicity was observed in nine patients, including one patient with SD (Figure 3A). Actigraphies detected a long time sleeping (TST > 10 h) in one patient, with an average TST for 7 days of 1,025 min.

In conclusion, sleep disorders in LGI1-encephalitis patients included insomnia, hypersomnia, RBD, PLMS, and OSAS.

All 27 patients showed good responses to immunomodulatory therapies including steroids, intravenous immunoglobulin (IVIG), and/or immunosuppressants. Their average hospital days stay was 21 (8–47) days. Two cases were followed up on PSGs without relapses. One of the two patients had SD during the acute phase. Her repeated PSG recording revealed identified sleep architecture and stable REM sleep (Figure 1B). She also had an increase of TST from 267 to 448.5 min, sleep efficiency from 49.8% to 56.9%, and REM sleep from 1.5 to 10.9%. An improvement in PSG recording was also observed in the other patient (TST from 430 to 462.5 min; sleep efficiency from 80.3% to 84.8%; and REM sleep from 13.6% to 15.0%).

### CASPR2 Antibody Associated-Diseases

Seven patients with Caspr2-Ab diseases underwent detailed sleep assessments. Four patients had co-occurrence of LGI1-Ab at low titer. The sex ratio was 5:2 (male:female). The median age was 59 years (range 24–70 years). All cases developed neuromyokymia, dysautonomia, and sleep disturbances. Four patients had cognitive impairment or seizures and fulfilled the criteria of Morvan's syndrome. Other presenting symptoms were psychiatric disorders (57.1%), memory impairment (42.9%), ataxia (28.6%), seizures (28.6%), hyponatremia (57.1%), and consciousness disorders (14.3%) (Table 1). CSF studies showed elevated protein in two patients (0.53–0.69 g/L) and elevated white cells in two patients (10–17 cells/μl). Cerebral MRIs were available in six patients with normal finding. Of four patients who underwent PET/CTs, two patients had abnormal metabolic patterns in the basal ganglia and medial temporal lobe. Three patients had EEG recordings. One was normal, one revealed epileptiform discharge, one had mild excessive slow waves, and none had tumors.

All seven (100%) patients had insomnia. Five patients had new concerns of limb movements. Four of these patients had dream enactment behavior during the acute phase. Excessive daytime sleepiness was presented in three patients (Table 1).

PSG recordings were available in five patients. Sleep onset latency was 13 (3.5–41.5) min. TST was 329.5 (167–377.5) min. Sleep efficiency was 61.7% (34.6–71.7%) of sleep period time (median 478.5, range 438–570 min). The percentages of the respective sleep stages were 27.5% for N1 (8.3–60.2%), 51.0 % for N2 (0.7–73.2%), 15.0% for N3 (0–34.6%), and 12.7% for stage REM (0–22.2%). LMS index was 53.3/h (range 41–120.5/h). LMS indexes were higher than the range of values in age-matched healthy sleepers in four patients (20). PSG results demonstrated PLMS in one patient and epileptiform discharges in another patient. None had RWA/RBD and OSAS.

PSG recording demonstrated SD in two patients (Figure 1C). The range of TST was from 167 to 354 min. The range of sleep efficiency was from 34.6 to 71.7%. One patient lacked typical REM sleep, whereas another patient had no N3 sleep. Both cases were diagnosed with Morvan's syndrome and had increased LMS (114–120.5/h). Agrypnia excitata was identified in one patient who had complex movements mimicking the contents of dreams and severe insomnia. PLMS were detected in the other patients.

Actigraphies were performed in five patients. Four (80%) artigraphies displayed continuous insomnia with marked diurnal and nocturnal motor activities (Figure 3B). None had a long time sleeping.

All seven patients with Caspr2-Ab diseases had improvements on immunotherapy. Their hospital days were 29 (27–45) days. One patient died of pneumonia during relapse. None had repeated PSG evaluations.

## Discussion

Sleep disorders are common in VGKC-Ab diseases presenting in 20–65% patients with LGI1-Ab encephalitis (21–24) and 22–68% patients with Caspr2-Ab-associated diseases (15, 25). In our study, nearly half of the patients had sleep disturbances, which was consistent with previous studies. Different forms of sleep disorders were observed in our study including insomnia, hypersomnia, RBD, PLMS, agrypnia excitata, and OSAS.

PSGs in patients with LGI1-Ab encephalitis demonstrated a decrease in TST, sleep efficiency, and the percentages of REM and N3 sleep stages but a marked increase in LMS index. Consistent with previous reports (8, 11, 26), we found that RBD and PLMS were more common in LGI1-Ab encephalitis than in Caspr2-Ab diseases. LGI1-Ab encephalitis is characterized by limbic system involvements. Previous studies demonstrated that the limbic system is related to dreams during REM sleep and that its connections with the brainstem are responsible for REM sleep atonia. The dysfunction of the limbic system could account for RBD (26, 27). All patients with LGI1-Ab encephalitis had limbic encephalitis.

We found PSG parameters did not change with frequencies of RWA/RBD, PLMS, FBDSs, or epileptiform discharges in patients with LGI1-Ab encephalitis. We suggest that the declines of TST, sleep efficiency, REM, and N3 sleep were caused by encephalitis but no other sleep disorders.

SD was detected in VGKC-Ab diseases and was characterized by sleep fragments and state-determining boundaries breakdown. PSG showed frequent shifts between wake, N1, and N2 sleep stages, and REMs intermixed with slow eye movements. Agrypnia excitata was identified in one patient with Morvan's syndrome. We also provided evidence for SD in LGI1-Ab encephalitis. Studies about SD in LGI1-Ab encephalitis were very limited. Only one case report described agrypnia excitata associated with LGI1-Ab encephalitis (28). We found that 23.8% of patients with LGI1-Ab encephalitis presented SD. These patients were more likely to have simple limb movements rather than complex movements. We also found decreased REM sleep, increased N1 sleep, and a trend of N3 sleep decline in SD patients. Other PSG data and clinical features did not change with SD.

Actigraphies results revealed continuous insomnia in VGKC-Ab-associated diseases. Animal experiments demonstrated that VGKC complexes are related to sleep regulation. VGKC dysfunction may be responsible for the increased motor drive and reduction in sleep time (29, 30). We found that continuous insomnia was more frequent in patients with Caspr2-Ab-associated diseases than LGI1-Ab encephalitis, which suggested that Caspr2-Ab cause more severe sleep disturbances. Actigraphy is an alternative sleep assessment for patients who could not complete PSG owing to severe insomnia. However, actigraphy could not recognize the sleep stages or identify sleep structure.

Hypersomnia was not rare in VGKC-Ab-associated diseases. Long sleeping time was detected in actigraphy recordings. The basal ganglia play an important role in sleep–wake regulation. Bilateral lesions were made in the striatum, which resulted in a significant reduction in time spent in wakefulness (31). PET/CT revealed that basal ganglia were commonly involved in patients with VGKC-Ab diseases. Hypothalamus dysfunction, resulting in orexin-A reduction, could also be a reason for hypersomnia and other sleep disorders (32).

All patients showed improvements with immunotherapy. Follow-up PSG evaluations demonstrated an increase in TST and sleep efficiency and re-identified sleep structure.

## Conclusions

We found that sleep disorders including insomnia, hypersomnia, RBD, PLMS, agrypnia excitata, and OSAS were commonly encountered in VGKC-Ab diseases. A decrease in TST and sleep efficiency and changes in the respective sleep stages were observed. Evidence of SD in patients with LGI1-Ab encephalitis showed that these patients are more likely to have simple limb movements. However, the small sample size of patients with LGI1-Ab encephalitis who had SD may have a bias on results of statistical analysis. Patients with Morvan's syndrome who could not complete PSG monitor due to severe insomnia were not included. There is a need for more works to better understand sleep disorders and SD in VGKC-Ab-associated diseases.

## Data Availability Statement

All datasets generated for this study are included in the article/Supplementary Material.

## Ethics Statement

The studies involving human participants were reviewed and approved by Ethics Committee of Peking Union Medical College Hospital. The patients/participants provided their written informed consent to participate in this study.

## Author Contributions

All authors listed have made a substantial, direct and intellectual contribution to the work, and approved it for publication.

## Conflict of Interest

The authors declare that the research was conducted in the absence of any commercial or financial relationships that could be construed as a potential conflict of interest.
