# Corrigendum: Sleep Disorders in Leucine-Rich Glioma-Inactivated Protein 1 and Contactin Protein-Like 2 Antibody-Associated Diseases

**DOI:** 10.3389/fneur.2020.607331

**Published:** 2020-10-30

**Authors:** Nan Lin, Honglin Hao, Hongzhi Guan, Heyang Sun, Qing Liu, Qiang Lu, Liri Jin, Haitao Ren, Yan Huang

**Affiliations:** Department of Neurology, Peking Union Medical College Hospital, Beijing, China

**Keywords:** sleep disorders, polysomnography, states dissociate, LGI1 antibody encephalitis, Caspr2 antibody-associated diseases

In the original article, there was a mistake in Figure 1 as published. The sleep histogram in part 1C was mistakenly copied from the sleep histogram 1B. The corrected Figure 1 appears below.

**Figure 1:**
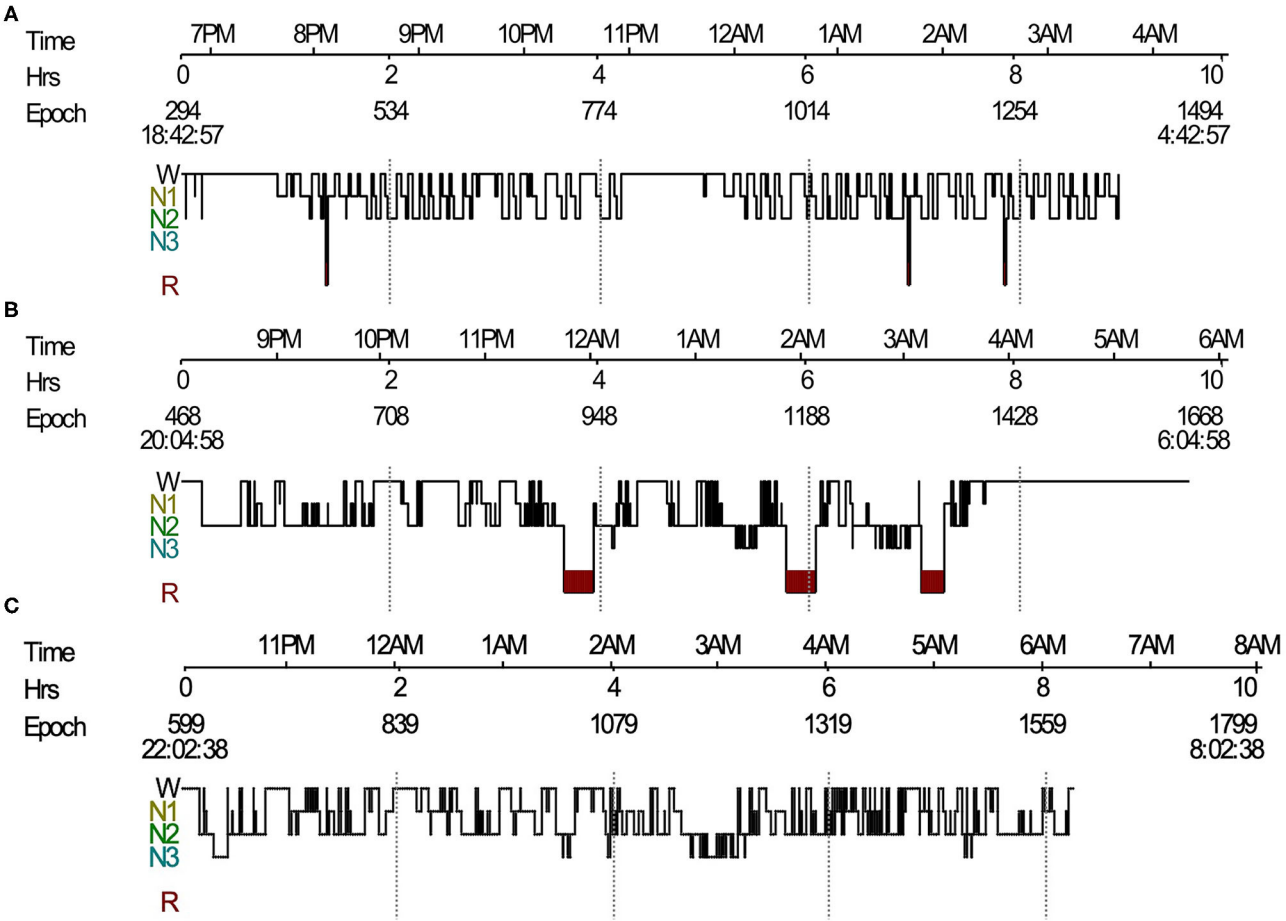
Sleep histogram. **(A)** Sleep histogram in a patient with LGI1-antibody encephalitis revealed severe impaired sleep structure, short REM sleep episodes mixed with NREM sleep, and no N3 sleep. **(B)** Follow-up PSG showed identified sleep architecture and stable REM sleep. **(C)** Sleep histogram in a Morvan's syndrome patient showed absence of REM sleep and frequent shifts between wake and NREM sleep. LGI1, leucine-rich glioma-inactivated protein 1; REM, rapid eye movement; NREM, non-rapid eye movement; PSG, polysomnography.

The authors apologize for this error and state that this does not change the scientific conclusions of the article in any way. The original article has been updated.

