# Supplementary material for: Sleep Disorders in Leucine-Rich Glioma-Inactivated Protein 1 and Contactin Protein-Like 2 Antibody-Associated Diseases
Source: Front Neurol. 2020 Jul 30;11:696. doi: 10.3389/fneur.2020.00696 (PMC7406672; doi:10.3389/fneur.2020.00696)
Supplement: Supplementary file 1 [file Table_1.DOCX]

Supplements

**Supplements 1**. The effects of FBDS, RWA, RBD, PLMS, epilepsy on the PSG parameters in LGI1 group.

|  | FBDS | | | RWA | | | RBD | | | PLMS | | | Epilepsy | | |
| --- | --- | --- | --- | --- | --- | --- | --- | --- | --- | --- | --- | --- | --- | --- | --- |
|  | Y  (n=14) | N  (n=7) | P | Y  (n=9) | N  (n=12) | P | Y  (n=5) | N  (n=16) | P | Y  (n=13) | N  (n=8) | P | Y  (n=10) | N  (n=11) | P |
| TST | 366.7  (267.0-467.5) | 342  (184.5-495.5) | 0.657 | 343.5  (184.5-466) | 366.7  (267-495.5) | 0.407 | 331.5  (184.5-466) | 366.7  (267-495.5) | 0.660 | 374.5  (273-495.5) | 332.0  (184.5-467.5) | 0.177 | 342.8  (93.4) | 393.2  (53.1) | 0.140 |
| Sleep efficiency | 71.5%  (50-92%) | 59%  (47-91%) | 0.406 | 68.0%  (47-92%) | 72.5%  (50-91%) | 0.854 | 70%  (47-92%) | 70%  (50-91%) | 0.842 | 72.0%  (54-91%) | 64.5%  (47-92%) | 0.425 | 65.4%  (15.1%) | 72.2%  (8.6%) | 0.218 |
| REM %TST | 8.7%  (0.4-23.7%) | 12.0%  (4.3-27.91%) | 0.185 | 15.0%  (5.1-27.9%) | 7.8%  (0.4-18.1%) | 0.243 | 17.2%  (6.2-27.9%) | 8.2%  (0.4-22.9%) | 0.040 | 9.9%  (0.4-22.9%) | 12.7%  (1.5-27.9%) | 0.744 | 13.6%  (11.3%) | 14.5%  (6.5%) | 0.830 |
| N3 %TST | 9.5%  (0-32.9%) | 11.4%  (0.3-26.1%) | 0.910 | 10.3%  (0-28.7%) | 8.9%  (0-32.9%) | 0.06 | 11.7%  (9.7-28.7%) | 8.3%  (0-32.9%) | 0.075 | 8%  (0-32.9%) | 10.5%  (0-28.7%) | 0.804 | 11.9%  (11.0%) | 13.0%  (11.2%) | 0.834 |
