# Supplementary material for: Sleep Disorders in Leucine-Rich Glioma-Inactivated Protein 1 and Contactin Protein-Like 2 Antibody-Associated Diseases
Source: Front Neurol. 2020 Jul 30;11:696. doi: 10.3389/fneur.2020.00696 (PMC7406672; doi:10.3389/fneur.2020.00696)
Supplement: Supplementary file 2 [file Table_2.DOCX]

VGKC antibodies, including LGI1 antibody and Caspr2 antibody, are related to a group of nervous system autoimmune disorders, and also one of the most common antibodies in autoimmune encephalitis patients. Sleep disorders are very common in people with VGKC antibody associated diseases. We set out the largest study to date on PSG characteristics in patients with LGI1 antibody and Caspr2 antibody associated diseases. According to previous studies, patients with Caspr2 antibody associated diseases, can presented very severe sleep disorders, like agrypnia excitata. PSG in patients with agrypnia excitiata showed complete breakdown of state-determining boundaries and sleep fragmentation, which is called status dissociates (SD). Our study confirmed agrypnia excitata presented in Caspr2 antibody associated diseases, and detected SD in patients with LGI1 antibody associated encephalitis. We first analyzed the data of patients with SD in LGI1 group, and found they were more likely to have simple limb movements, with circadian rhythm remained.
